# The prognostic value of postoperative blood glucose in non-diabetic patients with rheumatic heart disease

**DOI:** 10.1186/s12872-019-01278-2

**Published:** 2019-12-17

**Authors:** Wan-zi Hong, Yu Wang, Hongjiao Yu, Xue-biao Wei, Danqing Yu, Chun-xiang Zhang, Ning Tan, Lei Jiang

**Affiliations:** 1 Department of Cardiology, Guangdong Cardiovascular Institute, Guangdong Provincial People’s Hospital, Guangdong Academy of Medical Sciences, Guangzhou, 510080 China; 2 Institute of Medical Sciences, School of Medical Sciences, University of Aberdeen, Foresterhill, Aberdeen, UK; 3 Guangdong Provincial Geriatrics Institute, Guangdong Provincial People’s Hospital, Guangdong Academy of Medical Sciences, Guangzhou, 510080 China; 4 School of Medicine, University of Alabama at Birmingham, Birmingham, AL US

**Keywords:** Rheumatic heart disease, Valve replacement surgery, Blood glucose, In-hospital death

## Abstract

**Background:**

Blood glucose (BG) is a risk factor of adverse prognosis in non-diabetic patients in several conditions. However, a limited number of studies were performed to explore the relationship between postoperative BG and adverse outcomes in non-diabetic patients with rheumatic heart disease (RHD).

**Methods:**

We identified 1395 non-diabetic patients who diagnosed with having RHD, and underwent at least one valve replacement and preoperative coronary angiography. BG was measured at admission to the intensive care unit (ICU) after surgery. The association of postoperative BG level with in-hospital and one-year mortality was accordingly analyzed.

**Results:**

Included patients were stratified into four groups according to postoperative BG level’s (mmol/L) quartiles: Q1 (< 9.3 mmol/L, *n* = 348), Q2 (9.3–10.9 mmol/L, *n* = 354), Q3 (10.9–13.2 mmol/L, *n* = 341), and Q4 (≥ 13.2 mmol/L, *n* = 352). The in-hospital death (1.1% vs. 2.3% vs. 1.8% vs. 8.2%, *P* < 0.001) and MACEs (2.0% vs. 3.1% vs. 2.6% vs. 9.7%, *P* < 0.001) were significantly higher in the upper quartiles. Postoperative BG > 13.0 mmol/L was the best threshold for predicting in-hospital death (area under the curve (AUC) = 0.707, 95% confidence interval (CI): 0.634–0.780, *P* < 0.001). Multivariate logistic regression analysis indicated that postoperative BG > 13.0 mmol/L was an independent predictor of in-hospital mortality (adjusted odds ratio (OR) = 3.418, 95% CI: 1.713–6.821, *P* < 0.001). In addition, Kaplan–Meier curve analysis showed that the risk of one-year death was increased for a postoperative BG > 13.2 (log-rank = 32.762, *P* < 0.001).

**Conclusion:**

Postoperative BG, as a routine test, could be served as a risk measure for non-diabetic patients with RHD.

## Novelty statement

Blood glucose is critical for patients undergoing surgery. This study aimed to investigate the effects of postoperative blood glucose and determine whether it could be a valuable factor for non-diabetes patients of rheumatic heart disease undergoing valve replacement surgery, at last, 1395 non-diabetic patients were included in this study, and postoperative BG was shown to be served as a risk-stratified measure for non-diabetic patients with RHD.

## Background

Rheumatic heart disease (RHD) is an abnormal autoimmune response caused by group A Streptococcus (GAS) bacterium. As a major burden in developing countries, RHD affects >34 million people, causing >345,000 deaths, and 10 million disability-adjusted life years (DALYs) lost per year [1]. Valve replacement surgery can effectively improve the patients’ quality of life. However, that surgical method wouldn’t be beneficial for all RHD patients, especially for aged patients [2]. Therefore, it is imperative to find effective predictors to find out these patients and pay more attention to improve postoperative survival.

Diabetes mellitus is a metabolic disease characterized by chronic hyperglycemia, and that is a worldwide public health problem, as well as a serious threat to human health. Currently, the prevalence of diabetes has dramatically increased. Compared with non-diabetic patients, diabetes can affect all the vital organs of the body, so that the risk of heart, brain, and peripheral vascular diseases was significantly increased [3]. In patients with New Delhi metallo-beta-lactamase (NDM), the risk of adverse events was linked to hyperglycemia [4]. A previous study has shown that preoperative hyperglycemia is an independent predictor of 30-day and 3-month mortality in valve replacement [5]. Therefore, blood glucose is important for patients with RHD. However, as a postoperative routine examination, a small number of studies have focused on postoperative blood glucose. Therefore, this study aimed to investigate the effects of postoperative blood glucose and determine whether it could be a valuable factor for RHD after valve replacement surgery for patients without diabetes.

## Methods

### Study design and population

**Fig. 1:**
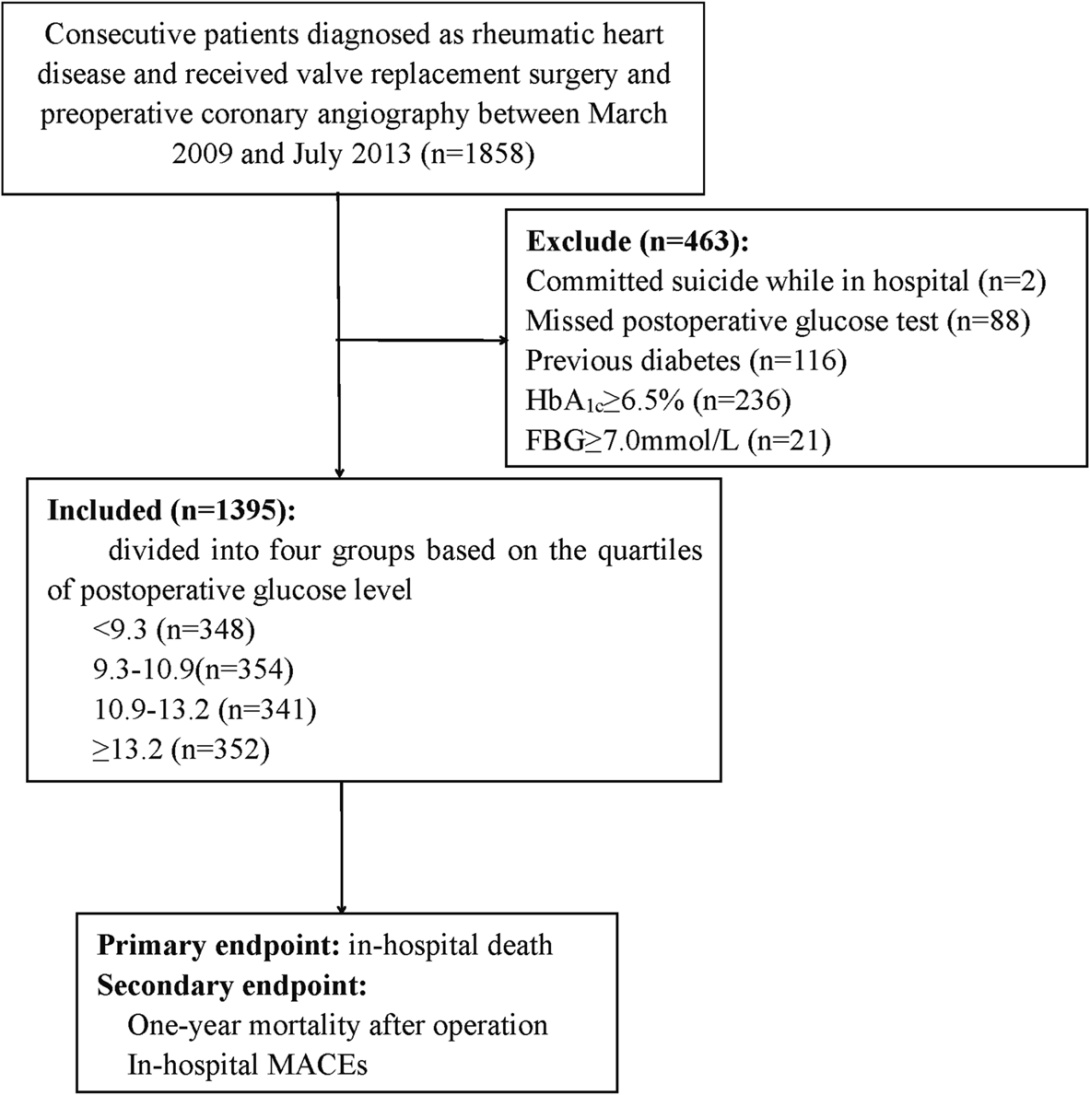
Flowchart of the population screened

A total of 1858 patients with RHD were consecutively screened in the Guangdong General Hospital (Guangzhou, China) between March 2009 and July 2013. All the patients with RHD received at least 1 valve replacement and preoperative coronary angiography. RHD was diagnosed according to previous acute rheumatic fever and/or symptom of precordial abnormalities and presence of heart murmur, and more importantly based on echocardiographic findings. Patients who committed suicide in hospital, or did not have a postoperative glucose data, or have diabetes were excluded from this study. Subjects with HbA1c ≥ 6.5% or fasting plasma glucose (FGB) ≥ 7.0 mmol/L were excluded as well. Finally, 1395 patients were eligible to be included in our analysis (Fig. 1). The primary endpoint was in-hospital all-cause mortality except for suicide during hospitalization, and the secondary endpoint was one-year mortality after operation and in-hospital major adverse clinical events (MACEs), which were defined as composite end points, such as death, renal failure with dialysis, and stroke.

This study was approved by the Ethics Committee of the Guangdong Provincial People’s Hospital, accompanying with a waiver of informed consent due to the retrospective study design. Oral informed consent was obtained from the patients or their close relatives by telephone, and was recorded by trained nurses during the follow-up period.

### Data collection and follow-up

Baseline clinical characteristics and laboratory data, in-hospital mortality, and the type of surgery were collected. Blood samples for evaluating glucose levels were collected at admission to the intensive care unit (ICU) after operation. The FBG was measured by using an autoanalyzer (Roche AG, Basel, Switzerland). Other venous blood samples for laboratory analysis were collected in the next morning after admission to the ICU before operation. Follow-up data were obtained from interviewing patients, family members, or primary care physicians by telephone or clinical records of hospital readmissions or outpatient interviews for a period of one year after operation.

### Statistical analysis

SPSS software version 13.0 (SPSS, Inc., Chicago, Illinois) was used for the analyses. Continuous data was presented as mean ± SD or medians and interquartile ranges, then compared by the ANOVA or Wilcoxon rank-sum test accordingly. Categorical data was presented as percentage and compared by χ^2^ or fisher test. Variables whose *p* value was less than 0.05 in univariate logistic regression analysis were included in the multivariable analysis. Kaplan–Meier curve analysis was performed to evaluated cumulative rate of one-year mortality and compared using the log-rank test. A value of *p* < 0.05 was considered significant.

## Results

### Patients’ clinical characteristics

**Fig. 2:**
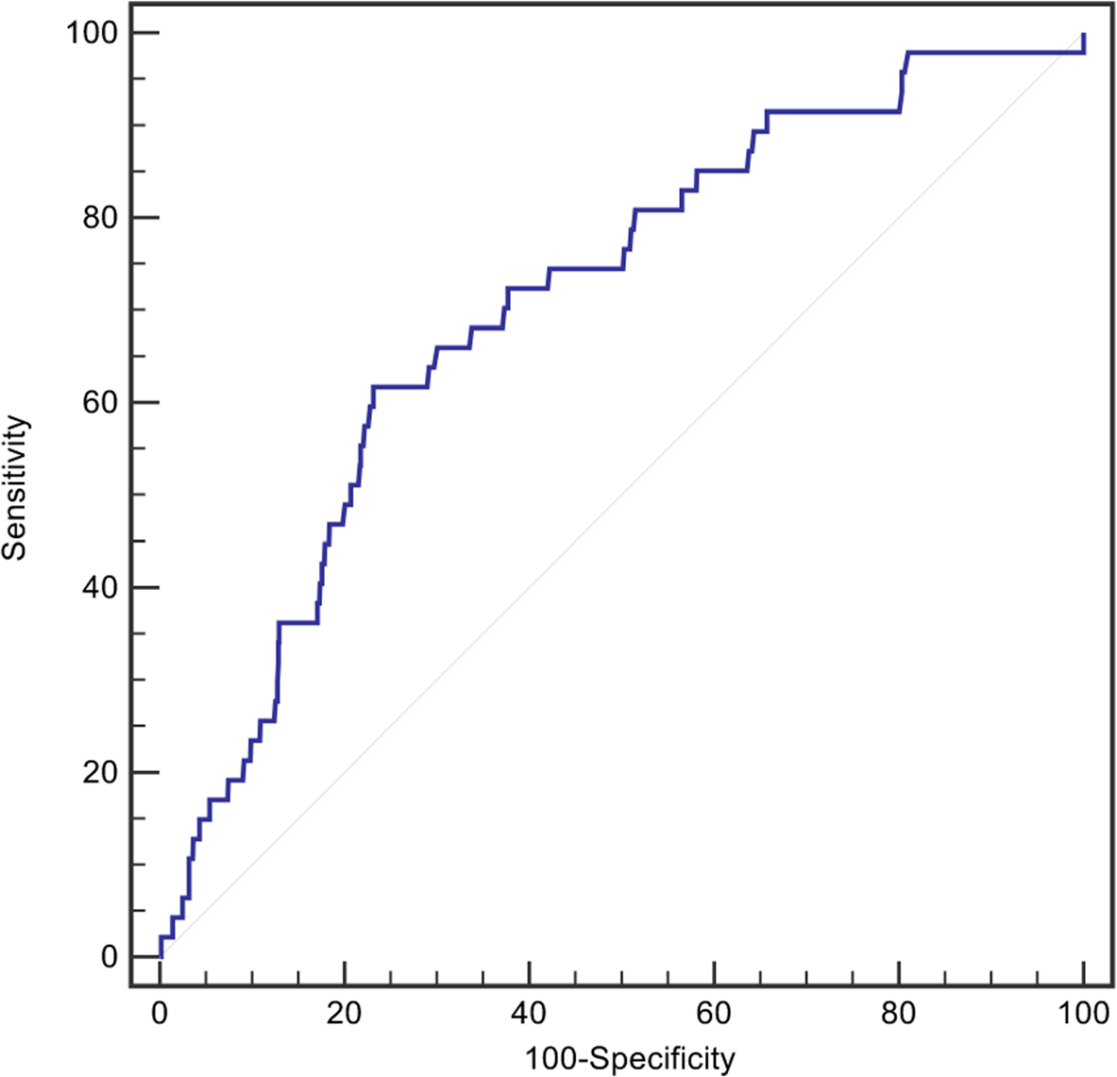
Receiver operating characteristic (ROC) curve of postoperative blood glucose for in-hospital death

Here, 1395 patients (female, 67.1%; male, 32.9%; age, 57 ± 6 years) were participated in this study, including 348 in Q1 group (< 9.3 mmol/L), 354 in Q2 group (9.3–10.9 mmol/L), 341 in Q3 group (10.9–13.2 mmol/L), and 352 in Q4 group (≥ 13.2 mmol/L) (Table 1). Patients with a high postoperative glucose level were significantly older (56.3 ± 5.7 vs. 57.2 ± 5.5 vs. 57.5 ± 5.7 vs. 58.0 ± 5.5, *P* = 0.001) and were more likely female (39.7% vs. 33.1% vs. 31.7% vs. 27.3%, *P* = 0.006). No significant differences were found among those 4 groups in the percentages of smoking, hypertension, mitral valve replacement, Tricuspid intervention, coronary artery bypass grafting (CABG), the level of serum creatinine, and Glycated hemoglobin. However, the percentages of NYHA > II, and Aortic valve replacement, the level of FBG, and lg C-eactive protein (lgCRP) were significantly higher in patients with a high postoperative glucose. In addition, the rate of left ventricular ejection fraction (LVEF) was lower in those patients. 47 (3.4%) patients died during the hospitalization, among which 4 (1.1%) were in Q1, 8 (2.3%) were in Q2, 6 (1.8%) were in Q3, and 29 (8.2%) were in Q4 (P<0.001). Furthermore, the incidence of in hospital MACEs (2.0% vs. 3.1% vs. 2.6% vs. 9.7%, *p* < 0.001) was significantly different among the four groups. Receiver operating characteristic (ROC) curve showed that postoperative BG > 13.0 mmol/L was the best threshold for predicting in-hospital death, accompanying with a sensitivity of 61.7% and specificity of 76.9% (area under the curve (AUC) = 0.707, 95% confidence interval (CI): 0.634–0.780, *P* < 0.001, Fig. 2).

**Table 1:** Patients’ baseline characteristics

Clinical variables	Postoperative BG level (mmol/L)				
	Quartile 1 (*n* = 348)	Quartile 2 (*n* = 354)	Quartile 3 (*n* = 341)	Quartile 4 (n = 352)	*P*
Age (year)	56.3 ± 5.7	57.2 ± 5.5	57.5 ± 5.7	58.0 ± 5.5	0.001
Males, n (%)	138 (39.7)	117 (33.1)	108 (31.7)	96 (27.3)	0.006
Smoking, n (%)	37 (10.6)	42 (11.9)	30 (8.8)	36 (10.2)	0.618
Hypertension, n (%)	29 (8.3%)	30 (8.5)	39 (11.4)	40 (11.4)	0.319
NYHA III-IV, n (%)	148 (42.5)	129 (36.4)	162 (47.5)	164 (46.6)	0.012
FBG (mmol/L)	4.7 ± 0.5	4.8 ± 0.6	4.9 ± 0.6	4.9 ± 0.6	<0.001
Postoperative glucose (mmol/L)	8.1 ± 1.0	10.1 ± 0.5	12.0 ± 0.7	15.8 ± 2.0	<0.001
Serum creatinine (umol/L)	83.4 ± 29.7	78.6 ± 22.3	79.6 ± 24.6	81.5 ± 25.7	0.069
lgCRP (mg/L)	0.5 ± 0.4	0.5 ± 0.4	0.5 ± 0.4	0.6 ± 0.4	0.006
Glycated hemoglobin (%)	5.8 ± 0.4	5.8 ± 0.4	5.8 ± 0.4	5.8 ± 0.4	0.222
LVEF (%)	61.7 ± 8.6	62.1 ± 8.6	62.5 ± 8.5	60.7 ± 10.3	0.047
Type of surgery					
Aortic valve replacement	170 (48.9)	163 (46.0)	189 (55.4)	198 (56.3)	0.015
Mitral valve replacement	331 (95.1)	318 (95.5)	314 (92.1)	326 (92.6)	0.174
Tricuspid intervention	270 (77.6)	275 (77.7)	254 (74.5)	271 (77.0)	0.730
CABG	13 (3.7)	12 (3.4)	14 (4.1)	20 (5.7)	0.445
In-hospital death	4 (1.1)	8 (2.3)	6 (1.8)	29 (8.2)	<0.001
In-hospital MACEs	7 (2.0)	11 (3.1)	9 (2.6)	34 (9.7)	<0.001

*NYHA* New York Heart Association, *FBG* Fasting blood glucose, *CRP* C-reactive protein, *LVEF* Left ventricular ejection fraction, *CABG* Coronary artery bypass grafting, *MACEs* Major adverse clinical events

In the univariable logistic regression analysis, the postoperative BG > 13.0 mmol/L was associated with in-hospital death (odds ratio (OR) = 4.666, 95% CI: 2.559–8.507, *P* < 0.001). The significant variables were age, NYHA III/IV, estimated glomerular filtration rate (eGFR) < 60 ml/min/1.73 m2, lgCRP, LVEF, TVR, and CABG. These variables were entered into a multiple logistic regression analysis, in which the results showed that postoperative BG > 13.0 mmol/L remained an in- dependent predictor of in-hospital mortality (adjusted OR = 3.418, 95% CI: 1.713–6.821; *P* < 0.001). In addition, it was revealed that the eGFR <60 ml/min/1.73 m2 (OR = 2.952, 95%CI: 1.311–6.645, *P* = 0.009) and TVR (OR = 3.855, 95%CI: 1.127–3.52, *P* = 0.032) were independently associated with in-hospital death (Table 2).

**Table 2:** Univariate and multivariate logistic analyses for in-hospital death

Clinical variables	Univariate Analysis			Multivariate Analysis		
	OR	95% CI	*P*	OR	95% CI	*P*
Age	1.1.06	1.054,1.160	<0.001	1.056	0.994,1.122	0.076
Female	0.714	0.394,1.293	0.266			
Smoking	0.796	0.282,2.251	0.668			
Hypertension	1.348	0.562,3.235	0.504			
NYHA III-IV	1.981	1.095,3.583	0.024	1.459	0.720,2.959	2.95
FBG	0.859	0.494,1.495	0.592			
Postoperative BG > 13.0 mmol/L	4.666	2.559,8.507	<0.001	3.418	1.713,6.821	<0.001
eGFR <60 ml/min/1.73 m^2^	3.568	1.796,7.088	<0.001	2.952	1.311,6.645	0.009
LgCRP	3.580	1.751,7.321	<0.001	2.162	0.968,4.826	0.060
Glycated hemoglobin						
LVEF	0.956	0.930,0.983	0.001	0.973	0.942,1.004	0.089
AVR						
MVR						
TVR	2.615	1.026,6.665	0.044	3.855	1.127,13.188	0.032
CABG	3.576	1.454,8.792	0.006	2.821	0.869,9.156	0.084

*NYHA* New York Heart Association, *eGFR* estimated glomerular filtration rate, *BG* Blood glucose, *CRP* C-reactive protein, *CABG* Coronary artery bypass grafting

**Fig. 3:**
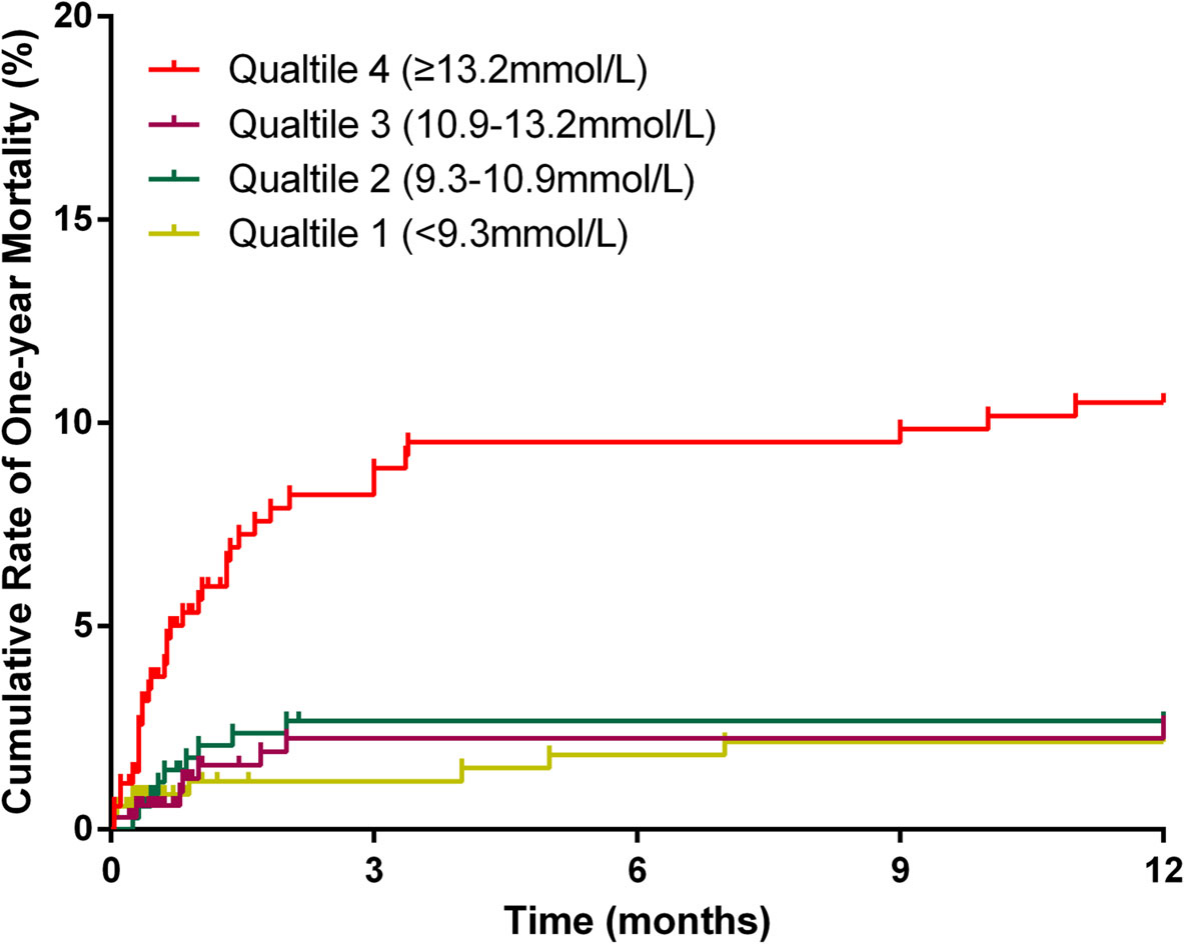
Cumulative rate of 1-year mortality for patients in 4 different groups of postoperative blood glucose

A total of 1248 (89.5%) patients completed one-year follow-up after surgery. However, during this period, 58 patients died. Result of Kaplan–Meier curve analysis is displayed in Fig. 3, illustrating the cumulative 1-year mortality among the four groups. The results suggested that the risk of death was increased for a postoperative BG > 13.2 (log-rank 32.762, *P* < 0.001) (Fig. 3).

## Discussion

Our study revealed that high postoperative BG level was associated with increased morbidity and mortality in RHD patients undergoing VRS, while preoperative BG level was not associated with those factors.

The mechanism of how an elevated postoperative BG level was associated with mortality has not been still fully understood. Thus, the following aspects should be taken into account.

Firstly, body glucose levels are regulated by the endocrine system. Postoperative acute hyperglycemia is regarded as the body’s stress response, and stress hyperglycemia complicated by cardiac surgery is clinically common, especially after cardiopulmonary bypass, the incidence rate reaches 95–100% [6]. Gandhi et al. suggested that when the BG level was greater than 100 mg/dl, the risk of various complications could be increased by 30% for every 20 mg/dl increase [7]. Thoracic surgery is a strong stimulus for the body, and when the variability of patient’s BG level is considerable, it will aggravate the damage caused by such stress reaction, that is equivalent to a “second strike” effect [8].

On the one hand, hyperglycemia is always associated with hyperlactatemia [9, 10], increasing morbidity and mortality in acute critical illness [11, 12]. A previous confirmed that lactate levels might appropriately reflect the severity of disease and organ failure, and also were independently associated with short-term mortality in critically ill patients with liver cirrhosis [13]. Although anaerobic glycolysis increases the substrate, however, suppressing hyperglycemia generating 2,3-diphosphoglycerate, absolute insulin secretion, or relatively insufficient may lead to an increase in plasma free fatty acid concentration, and fatty acid can increase the myocardial oxygen consumption.

On the other hand, BG concentration can influence the function of immune system. The inflammatory response is closely associated with the prognosis of surgery and has been demonstrated in a large number of studies [14, 15]. Studies have shown that inflammation affects wound healing and leads to an increase in postoperative mortality [16]. A variety of inflammatory mediators have also been shown as predictors of postoperative risk [17]. Researches have shown that plasma levels of interleukin 8 (IL-8) and C-reactive protein (CRP) are higher in patients with hyperglycemia than in patients with normal BG levels [18, 19], thereby reducing T cell expression and the body’s immune response [20–22]. All the above-mentioned reasons may explain that the immune response may be the cause of acute hyperglycemia, leading to a poor prognosis in patients undergoing cardiac surgery.

This study had some limitations. First, as this was a retrospective analysis based on prospectively collected data, some confounding might have affected the results. Second, the primary endpoint was all-cause mortality except for suicide which reduced the ability to fully evaluate the causes of death and consequently accurately compare the “cause of death” outcomes from this study with those of other populations.

## Conclusions

In conclusion, we demonstrated that with the increase of postoperative BG, the prognosis of patients is worse. BG is able to accurately predict in-hospital death and mortality in patients with RHD after valve replacement surgery. Furthermore, we found that BG > 13.0 mmol/L is the optimum threshold for predicting in-hospital mortality.

## Data Availability

the findings. The datasets used and/or analysed during the current study are available from the corresponding author on reasonable request.

## Abbreviation

AUC: Area under the curve
BG: Blood glucose
CABG: Coronary artery bypass grafting
CI: Confidence interval
CRP: C-reactive protein
eGFR: Estimated glomerular filtration rate
FBG: Fasting blood glucose
ICU: Intensive care unit
IL-8: Interleukin 8
LVEF: Left ventricular ejection fraction
MACEs: Major adverse clinical events
NYHA: New York heart association
OR: Odds ratio
RHD: Rheumatic heart disease
ROC: Receiver operating characteristic
VRS: Valve replacement surgery
